# Short‐Term Interactive Effects of Temperature and Air Pollution on Cardiovascular Visits: A Machine‐Learning Study in Hohhot, China

**DOI:** 10.1155/crp/9938043

**Published:** 2026-11-10

**Authors:** Xinyu Mao, Tao Wang, Xin Jiao, Meilian Deng, Chunyi Xin, Shuting Lü, Yiqi Wang, Yujie Ren, Li Qi, Weiping Liu

**Affiliations:** ^1^ School of Public Health Inner Mongolia Medical University Hohhot China immu.edu.cn; ^2^ College of Medical, Veterinary & Life Sciences University of Glasgow Glasgow UK gla.ac.uk; ^3^ Department of Public Health Inner Mongolia People’s Hospital Hohhot China nmgyy.cn

**Keywords:** air pollution, ambient temperature, cardiovascular disease, interaction effects, machine learning

## Abstract

**Introduction:**

The synergistic effects of ambient temperature and air pollutants pose a substantial public health burden related to cardiovascular disease (CVD). However, their high dimensionality, nonlinearity, and temporal lag structures make them difficult to characterize with conventional statistical methods.

**Methods:**

We developed a machine‐learning framework to evaluate the short‐term interactive effects of ambient temperature and six major air pollutants (PM_2_._5_, PM_10_, SO_2_, NO_2_, CO, and O_3_) on CVD visits. Using 508,077 records from tertiary hospitals in Hohhot, China (2016–2024), we matched daily CVD visits to these environmental data. A high‐order polynomial ridge regression modeled nonlinear exposure interactions, quasi‐Poisson regression captured lag structures, and a random forest assessed overall model performance.

**Results:**

High temperatures significantly amplified the cardiovascular risks of PM_2_._5_ and SO_2_ exposure, with PM_2_._5_ showing a cumulative percentage change of 30.96% at lag07 (95% CI: 30.86%–31.05%, *p* < 0.001). Conversely, the adverse effects of PM_10_, NO_2_, CO, and O_3_ were more pronounced under low temperatures. The random forest model achieved high predictive accuracy (cross‐validated *R* ^2^ = 0.741).

**Conclusion:**

Air pollution and temperature jointly influence CVD health risks. Machine learning improves characterization of these complex relationships. These findings support the development of early warning systems and targeted interventions for CVD prevention under varying temperature conditions.

## 1. Introduction

In recent years, air pollution has become an increasingly serious global concern. According to the World Health Organization’s air quality database, approximately 99% of the global population lives in areas where air quality does not meet recommended guidelines [1]. Air pollution has emerged as a major environmental health threat, particularly due to its substantial contribution to the burden of cardiovascular disease (CVD) [2]. CVD accounts for approximately 17.9 million deaths annually, representing about one‐third of all deaths worldwide [3]. Epidemiological evidence consistently indicates that short‐term exposure to air pollution can trigger acute cardiovascular events, including myocardial infarction, stroke, and arrhythmia [4–6]. Mechanistically, particulate matter and gaseous pollutants may act synergistically through pathways such as oxidative stress, inflammatory responses, and vascular endothelial dysfunction, thereby exacerbating cardiovascular damage [7, 8].

However, the magnitude of these effects varies across regions, pollutant types, and climatic conditions, indicating substantial heterogeneity in their health impacts. Furthermore, a growing body of evidence suggests that ambient temperature can significantly modify the cardiovascular effects of air pollutants. Multinational and multicenter studies have shown that high temperatures can amplify the cardiovascular risks associated with PM_2_._5_, whereas low temperatures may enhance the adverse effects of gaseous pollutants such as NO_2_ and CO [9]. Some studies have further reported that, compared with warmer seasons, the effects of NO_2_ and CO on CVD visits increase by approximately 20%–30% during colder periods [10].

However, several limitations remain in the existing literature. First, most studies have been conducted in regions with relatively stable temperature patterns, with limited attention given to populations exposed to substantial fluctuations in both high and low temperatures. Second, previous studies have largely relied on single‐pollutant or low‐dimensional models, which are insufficient for simultaneously capturing nonlinear relationships, multipollutant interactions, and lagged effects. As a result, the complex and dynamic interplay between multipollutant exposure and temperature cannot be fully characterized. In the context of large temperature variability and coexposure to multiple pollutants, the patterns of temperature–pollution interactions on CVD visits remain inadequately understood. To address these gaps, this study focuses on Hohhot, China, a region characterized by frequent air pollution episodes and substantial temperature variability [11], providing a suitable setting to investigate temperature–pollution interactions. We developed a machine learning–integrated analytical framework that employs ridge regression to model high‐order nonlinear interactions between temperature and pollutants, quasi‐Poisson regression to estimate short‐term lag effects, and random forest modeling to assess robustness. This approach aims to systematically evaluate the short‐term effects of temperature–pollutant interactions on CVD visits and to identify their potential nonlinear and lagged characteristics.

## 2. Materials and Methods

### 2.1. Case Data

The consultation data used in the study were obtained from the hospital case reporting system in Hohhot City, Inner Mongolia Autonomous Region, China. These data included daily outpatient and emergency department records from multiple clinical departments (including cardiology clinics, specialized cardiovascular clinics, cardiovascular surgery, and emergency departments) from 30 April 2016 to 30 September 2024. Extracted variables comprised fundamental patient information, including consultation date, primary diagnosis, gender, and age. Cases were classified and screened based on the 10th edition of the International Classification of Diseases (ICD‐10). The following six categories of CVD were included: hypertensive disease (I10–I15), ischemic heart disease (I20–I25), pulmonary heart disease (I26–I28), rheumatic heart disease (I00–I09), cases with CVD symptoms without a precise diagnosis (R00–R09), and other types of heart disease (I30–I52) [12].

### 2.2. Atmospheric Pollutants and Meteorological Information

Data on atmospheric pollutants were obtained from the China Air Quality Online Monitoring and Analysis Platform (https://www.aqistudy.cn/), covering daily pollutant concentrations in Hohhot from 30 April 2016 to 30 September 2024. The pollutants included PM_2_._5_ (µg/m^3^), PM_10_ (µg/m^3^), SO_2_ (µg/m^3^), NO_2_ (µg/m^3^), CO (mg/m^3^), and O_3_ (µg/m^3^). Notably, O_3_ concentrations were represented by the daily maximum 8‐h average, while the remaining pollutants were reported as 24‐h averages.

Meteorological data were retrieved from the China Meteorological Science Data Sharing Service (https://data.cma.cn/), provided by the China National Ground International Exchange (CNGIE). The dataset included daily mean air temperature (°C). Based on the distribution of daily mean temperatures over the study period, temperatures at or below the 25th percentile were categorized as low temperatures, while those at or above the 75th percentile were designated as high temperatures.

### 2.3. Statistical Methods

All data processing and model development were conducted using Python Version 3.12.0. Descriptive statistics were performed on case data, pollutant concentrations, and daily mean temperatures. Spearman correlation analysis was performed to evaluate the relationships between air pollutants and meteorological variables, serving as an initial screening for potential multicollinearity.

To capture the nonlinear interaction between daily mean air temperature *(T)* and pollutant concentration *(P)* on CVD visits *(Y)*, we generated a multivariate polynomial feature matrix *Φ*_*k*_(*T, P*) that contains all main effects and interaction terms up to degree *k* (*k* = 2–5). For each pollutant, five‐fold cross‐validation (random seed = 42) was used to determine the optimal degree *(k∗)* by maximizing the mean cross‐validated coefficient of determination (*R* ^2^) penalized by 0.005 × *k*, thereby balancing goodness of fit and model parsimony. Weekend, public‐holiday, COVID‐19 period, long‐term trend, and seasonal indicators were included to adjust for temporal confounding. Ridge regression with an *L*_2_ penalty (*α* = 1.0) was then fitted to

(1) Ŷ=Φk∗T,P,Confoundersβ,

where Ŷ denotes the predicted daily visit count and β the coefficient vector [13]. The *L*_2_ regularisation alleviates multicollinearity among higher‐order terms and prevents overfitting [14].

Based on six types of pollutants and daily mean air temperature data, two lag structures were defined: a single‐day lag (lag0–lag7) and a cumulative lag (lag01–lag07), where lag0 refers to the day of the clinic visit. On this basis, an interaction term was introduced for each pollutant and concurrent temperature to examine how temperature modifies the health impacts of each pollutant. All main effect terms and interaction terms were Z‐score standardized using the following formula:

(2) Zt=Xt−μσ,

where *X*_*t*_ is the original value of a given variable on day *t*, *μ* and *σ* denote the mean and standard deviation of that variable, respectively, and *Z*_*t*_ is its standardized value.

The standardized variables were included in a quasi‐Poisson regression model for estimating the effect of the temperature–pollutant interaction term on daily CVD visits. The model structure was as follows:

(3) logEYt=α+β1Zi,tlag+β2ZT,t+β3Zi,tlag×ZT,t+St,

where *E*[*Y*_*t*_] denotes the expected number of visits on day *t*; *α* represents the intercept; *β*_1_, *β*_2_, and *β*_3_ are the regression coefficients for pollutant exposure, ambient temperature, and their interaction, respectively; $Z_{i,t}^{\left(\text{lag}\right)}$ represents the standardized value of the *ith* pollutant within the specified lag window; *Z*_*T*,*t*_ refers to the standardized value of temperature; and *S(t)* represents a natural spline function used to control for the influence of confounding factors. The statistical significance level was set at 0.05.

The incidence rate ratio (*I* *R* *R* = *e* ^*β*^) and its 95% confidence interval were calculated based on the corresponding regression coefficient *β* and subsequently transformed into the percentage change (PC = (IRR − 1) × 100%). The PC expresses the estimated percentage change in CVD visits for the exposure contrast represented by the corresponding regression coefficient. Its interpretation depends on the scaling of the main effect or interaction term; PC > 0 indicates a positive association, whereas PC < 0 indicates a negative association.

To test the robustness of the main model, a random forest regression model was implemented using the scikit‐learn library in Python. The input variables included lagged, cumulative, and interaction terms for each pollutant and temperature, with the number of CVD visits as the output variable. Potential confounding factors were also controlled for in this model. The model hyperparameters were optimized by RandomizedSearchCV (5‐fold cross‐validation), including the number of trees (*n_estimators* = 215), the maximum depth (*max_depth* = 19), and the number of samples of the smallest leaf node (*min_samples_leaf* = 4) [15]. Model performance was evaluated using *R* ^2^ and the mean squared error (MSE). Additionally, learning curves were analyzed to evaluate potential overfitting or underfitting.

## 3. Results

### 3.1. Descriptive Analysis of Case Data

A total of 508,077 CVD outpatient and emergency department visits were included in this study, with a nearly equal distribution of male and female patients (Table 1). Approximately 75% of the cases occurred in individuals aged 48 years and above, indicating that middle‐aged and older adults were the most affected group. Regarding diagnostic categories, the most common diagnosis was CVD symptoms without a definitive diagnosis (30.69%), followed by hypertensive disease (29.17%).

**TABLE 1:** Descriptive statistics of CVD cases.

Variables		*N*
Diagnostic types	R00–R09	155,879
	I10–I15	148,190
	I20–I25	144,988
	I30–I52	55,185
	I26–I28	2148
	I00–I09	1687

Gender	Male	254,887
	Female	253,190

Age	Mean	56.78
	Minimum	0.13
	P25	48
	P50	58
	P75	67
	Maximum	111
	SD	14.67

Total		508,077

*Note:* Age values are in years. P25, P50, and P75 denote the 25th, 50th (median), and 75th percentiles, respectively.

Abbreviation: SD, standard deviation.

### 3.2. Descriptive and Correlation Analyses of Atmospheric Pollutants and Meteorological Factors

According to the descriptive statistics (Table 2), the overall concentration levels were generally consistent with the daily average limit of the Class II standard of the Chinese Ambient Air Quality Standards (GB 3095–2012), which establishes pollutant‐specific concentration limits for residential and urban areas. However, exceedances of PM_2_._5_, PM_10_, and O_3_ concentrations were observed during certain periods, particularly for PM_10_, which greatly exceeded 150 μg/m^3^ during peak periods. SO_2_, NO_2_, and CO concentrations were generally below their respective standard limits although some values approached or reached the critical thresholds. The daily mean air temperature ranged from −26.78°C to 29.39°C, indicating substantial seasonal variation.

**TABLE 2:** Descriptive statistics of air pollutants and temperature.

Variable	Mean	SD	Min	P25	P50	P75	Max	National limit	Exceed days	Exceedance rate (%)
PM_2_._5_ (μg/m^3^)	32.80	28.97	1	14	24	41	443	75	250	8.13
PM_10_ (μg/m^3^)	74.87	67.64	4	36	58	96	974	150	225	7.31
SO_2_ (μg/m^3^)	13.89	11.33	4	7	10	16	141	150	0	0
NO_2_ (μg/m^3^)	34.37	16.63	4	22	31	44	96	80	37	1.20
CO (mg/m^3^)	0.82	0.52	0.2	0.5	0.7	1	3.8	4	0	0
O_3_ (μg/m^3^)	95.38	40.05	10	66	93	122	236	160	194	6.31
Temperature (°C)	8.38	12.55	−26.78	−2.17	10.61	19.44	29.39	—	—	—

*Note:* Since air temperature has no corresponding limit value, it is indicated with “—”. Exceedance rate (%) = (Exceedance Days ÷ Total Observation Days) × 100. P25, P50, and P75 denote the 25th, 50th (median), and 75th percentiles, respectively.

Abbreviation: SD, standard deviation.

Spearman analysis (Figure 1) showed significant positive intercorrelations among PM_2_._5_, PM_10_, SO_2_, NO_2_, and CO (*r* = 0.38–0.85, *p* < 0.01). Conversely, each of these five pollutants exhibited significant negative correlations with both O_3_ and the daily mean air temperature, while O_3_ and temperature demonstrated a significant positive correlation (*p* < 0.01).

**FIGURE 1:**
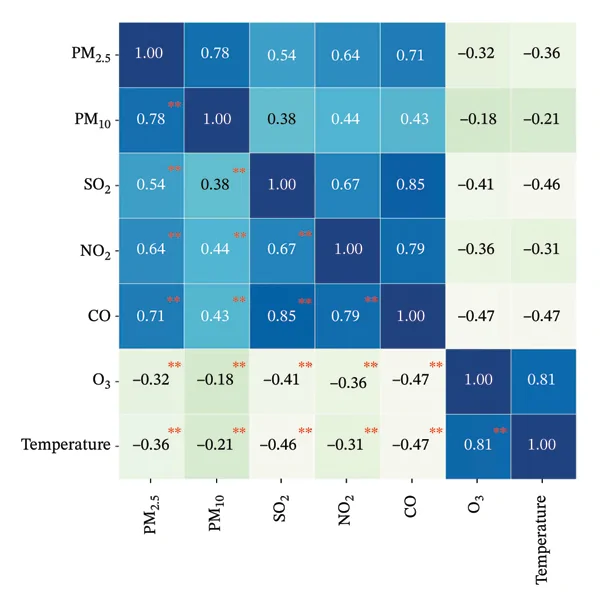
Spearman correlation matrix between the six air pollutants and daily mean air temperature. The color intensity represents the strength of the correlation (blue indicates positive correlation; green indicates a negative correlation). ^∗^ *p* < 0.01 and ^∗∗^ *p* < 0.001.

### 3.3. Atmospheric Temperature–Pollutant Interaction Effects

The interaction terms exhibited different scatterplot patterns across temperature ranges (Figure 2). PM_2_._5_, PM_10_, and NO_2_ demonstrated symmetrical scatter patterns, with PM_10_ showing more pronounced clustering under high‐temperature conditions. In contrast, the interaction values of SO_2_ and CO were predominantly concentrated in the low‐temperature range, and more active temperature–pollutant interactions were observed during cold conditions. The interaction term for O_3_ showed a noticeable increase with rising temperature, exhibiting a near‐exponential upward trend.

**FIGURE 2:**
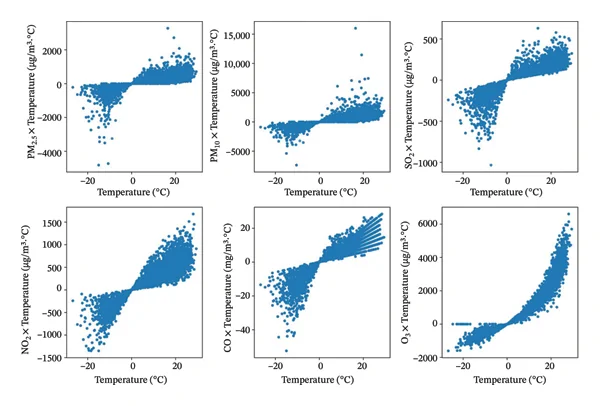
Scatter plots of the temperature–pollutant interaction terms against daily mean air temperature for the six air pollutants. In this figure, the temperature–pollutant interaction terms represent the unstandardized products of daily mean air temperature and pollutant concentrations, rather than model‐estimated interaction effects.

### 3.4. Joint Effects of Temperature and Air Pollutants on CVD Visits

Figure 3 shows the joint response surfaces of CVD visits to temperature and pollutant concentration. For PM_2_._5_ and SO_2_, CVD visits tend to increase with both temperature and concentration, with higher levels observed under conditions of elevated temperature and concentration. For PM_10_, NO_2_, and CO, higher visit counts are mainly observed under conditions of low temperature and high concentration, and this pattern becomes less pronounced as temperature increases, resulting in flatter response surfaces. In contrast, the response surface for O_3_ shows a distinct pattern, with higher CVD visits distributed over a broader region rather than concentrated at a single peak.

**FIGURE 3:**
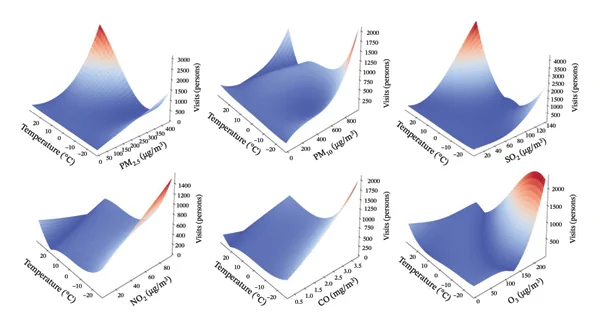
Three‐dimensional response surfaces of temperature, pollutant concentration, and CVD visits. Warmer colors and higher surface elevations represent higher numbers of visits.

### 3.5. Lagged Effect of Air Temperature–Pollutant Interaction on CVD Visits

Figure 4 demonstrates that the interaction term between temperature and pollutants had a statistically significant effect on daily CVD visits within the 0–7‐day lag interval (all 95% CIs did not cross 0, *p < *0.001). The interaction effect of PM_2_._5_ showed the strongest positive association, with a PC of 30.90% at lag0. The single‐day effect decreased slightly by 0.01–0.02 percentage points with each additional lag day, whereas the cumulative PC increased gradually, reaching 30.96% at lag07. The risk effect of SO_2_ was also positive but relatively small in magnitude (lag0 PC = 12.20%) and exhibited a decreasing trend across the entire lag window with slight and stable changes. In contrast, CO was associated with a consistently negative effect (lag0 PC = −29.30%), characterized by a gradual weakening of the single‐day lag effect by approximately 0.07%–0.08% per day. The cumulative effect remained stable throughout the period, ranging from −29.42% to −29.39%. The single‐day effects of NO_2_ and PM_10_ exhibited minimal fluctuations, with PC variations of < 0.02%. Their cumulative PCs slightly decreased to −15.31% and −15.17%, respectively. The single‐day lagged effects of O_3_ showed a relatively greater increase over time, increasing from approximately −6.78% at lag0 to an absolute increase of approximately 0.13%–0.14% at lag7. Simultaneously, the cumulative effect became slightly stronger, reaching approximately −6.92% at lag07.

**FIGURE 4:**
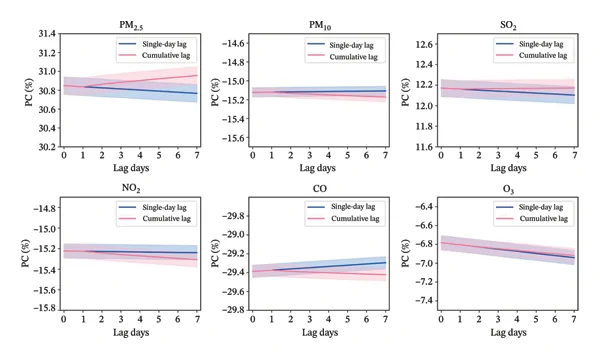
Single‐day and cumulative lagged 0–7‐day effects of the temperature–pollutant interaction terms on CVD visits. Shaded areas indicate the 95% confidence intervals (95% CIs). Percentage change (PC) denotes the estimated percentage change in CVD visits associated with the modeled temperature–pollutant interaction term for each specified lag window.

### 3.6. Robustness Analysis Based on Random Forest Model

The random forest model achieved an MSE of 1834.24 on the training set and 1982.50 on the independent test set. The *R* ^2^ reached 0.741. As the training sample size increased, the learning curve showed a gradual reduction in the gap between training and validation performance, with no evidence of overfitting or underfitting (Figure S1). The predicted versus observed values were highly consistent along the 1:1 reference line, with only a few outliers observed (Figure S2).

## 4. Discussion

This study employed a machine‐learning approach to systematically evaluate the interactions between ambient temperature and air pollutants, as well as their lagged effects. The results indicate that ambient temperature may modify the associations between air pollutant exposure and CVD visits. High temperatures were associated with stronger adverse associations of PM_2_._5_ and SO_2_, with PM_2_._5_ showing the most pronounced increase in CVD visit volume during the 7‐day cumulative lag period. In contrast, PM_10_, NO_2_, CO, and O_3_ exerted stronger risk effects under cold conditions. This temperature‐dependent modulation pattern has been consistently supported by a growing body of domestic and international evidence. For example, case‐crossover analyses in Shenzhen [16], time‐series research in Urumqi [17], and a multicenter case‐crossover study in the United States [18] have all confirmed that heatwaves and PM_2_._5_ act synergistically to increase the risk of CVD mortality or hospitalization significantly. A multinational study has also reported joint associations of heat and several ambient air pollutants with mortality [9]. Regarding NO_2_ and O_3_, several studies have reported stronger health effects during winter, indicating typical cold sensitivity [19, 20]. Additionally, a regional study in northern China reported that ambient temperature modified the association between O_3_ exposure and mortality, including stronger associations under some extreme‐temperature conditions [21].

Furthermore, from the perspective of biological mechanisms, the efficiency of airway cilia clearance decreases while mucus viscosity increases under cold conditions. This facilitates the deposition of NO_2_ and rapidly activates nicotinamide adenine dinucleotide phosphate oxidase, triggering oxidative stress and inhibiting nitric oxide production. Consequently, this process damages the vascular endothelium and reduces heart rate variability (HRV) [22, 23]. Meanwhile, O_3_ induces autonomic disorders through the activation of Transient Receptor Potential Ankyrin 1 (TRPA1) and Transient Receptor Potential Vanilloid 1 (TRPV1) ion channels and neurogenic inflammation. Low temperatures increase the excitability of these channels and weaken the airway barrier, potentially exacerbating the adverse biological effects of O_3_ exposure [23, 24]. Additionally, cold exposure triggers peripheral vasoconstriction, accompanied by increased blood viscosity, which synergistically amplifies the oxidative stress‐inflammation cascade, thereby accelerating the onset of acute ischemic or arrhythmic events [25].

The interaction between PM_2.5_ and SO_2_ under high temperatures became evident on the day of exposure and continued to build in subsequent days. This “long‐tailed” lag is consistent with recent multicity evidence showing that the mortality risk attributable to PM_2.5_ persists for several days and is magnified during hot weather [9, 26]. The mechanism may involve PM_2_._5_ and SO_2_ triggering inflammatory mediators such as interleukin‐6 and C‐reactive protein, which peak at 24–72 h and extend their half‐life due to physiological responses to high temperatures, including dehydration and hemoconcentration, thereby sustaining vascular endothelial activation [27].

It is worth noting that for PM_10_, NO_2_, CO, and O_3_, the direction of the associations differed between the ridge regression model and the quasi‐Poisson model. The ridge regression focused on high‐order interactions between temperature and pollutants under extreme exposure scenarios, making it more suitable for identifying amplified health risks in “extreme weather + high pollution” conditions. In contrast, the quasi‐Poisson model estimated the main effects based on overall average exposure levels, which more closely reflect population‐level responses under typical environmental conditions. The “protective” main effects observed in the quasi‐Poisson model should not be interpreted as indicating that these pollutants are beneficial to cardiovascular health. Rather, they likely reflect a statistically negative association influenced by multiple confounding factors. The overall concentrations of PM_10_, NO_2_, CO, and O_3_ in the study area were relatively low and rarely exceeded the national Grade II air quality standards. Under such “low‐concentration exposure” scenarios, the actual health effects of these pollutants may be minimal or may follow a U‐shaped or J‐shaped nonlinear dose–response relationship.

The machine‐learning framework introduced in this study showed good performance in capturing complex exposure–response relationships. The random forest model explained 74.1% of the temporal variation in CVD visit counts, and its predictions closely aligned with the results of both ridge regression and quasi‐Poisson models, further validating the robustness of the observed temperature–pollutant interactions. Compared to traditional linear approaches, ensemble learning algorithms offer greater capacity in handling nonlinear exposure patterns and high‐dimensional interaction structures [27–29]. They can also enhance model transparency through tools such as feature importance evaluation and other explainable machine‐learning techniques [30]. Previous studies have consistently shown that ensemble learning models outperform classical regression in both accuracy and adaptability in pollutant forecasting and environmental health risk modeling tasks [27–32].

This study has several limitations. First, it focused on short‐term exposure effects and did not address long‐term health outcomes. In addition, the data were derived from a single city, and the generalizability of the findings requires cautious validation before broader regional application. Furthermore, the ambient air pollution data used in this study were based on city‐level averages, which may not accurately reflect individual‐level exposure and could introduce exposure misclassification bias. Future research should expand the scope by incorporating multidimensional analyses and integrating multicenter datasets to enhance regional representativeness. In addition, further studies may consider stratified analyses by age and sex, as well as the integration of socioeconomic and demographic factors, to better characterize population heterogeneity in exposure–response relationships. Mechanistically, toxicological experiments may help elucidate underlying biological pathways, while molecular epidemiological data can support the construction of multilevel environment–biology–disease association models. Furthermore, machine‐learning approaches could be employed to simulate health burdens under extreme climate scenarios, and comparative studies between northern and southern regions may provide cross‐regional evidence to inform adaptive public health strategies in the context of climate change.

## 5. Conclusions

In summary, this study demonstrates that air pollution and ambient temperature jointly influence CVD health risks, exhibiting distinct modification patterns under different temperature conditions. By integrating machine‐learning methods with traditional epidemiological models, this approach more effectively captures the complex nonlinear and lagged interactions between temperature and multiple air pollutants, thereby providing a novel analytical perspective for assessing health effects under multiexposure scenarios. These findings highlight the importance of considering the effect modification of the association between air pollution and CVD visits by ambient temperature in health risk assessment and disease prevention, particularly in regions with substantial temperature variability. Furthermore, they provide scientific evidence to support the development of meteorology‐based air pollution health warning systems, the optimization of public protection measures under extreme temperature conditions, and the formulation of differentiated and dynamic strategies for CVD prevention and control, thereby enhancing the precision and timeliness of public health interventions.

## Author Contributions

Xinyu Mao led the study conceptualization, coordinated data collection and investigation, contributed to resource management and model validation, and drafted and revised the manuscript. Tao Wang assisted in study design, methodology development, and critical manuscript revision. Li Qi contributed to conceptualization, performed formal analysis, and participated in drafting. Meilian Deng, Yujie Ren, and Xin Jiao contributed to data curation and original draft writing. Chunyi Xin, Shuting Lü, and Yiqi Wang participated in data collection and field investigation. Weiping Liu supervised the project, contributed to model validation and manuscript revision, and served as the corresponding author.

## Funding

This study was supported by the Natural Science Foundation of Inner Mongolia Autonomous Region (Grant No. 2022MS08083).

## Ethics Statement

Ethical approval for this study was obtained from the Medical Ethics Committee of the Inner Mongolia Autonomous Region People’s Hospital (Approval No. 202517206L; approved on June 12, 2025).

## Conflicts of Interest

The authors declare no conflicts of interest.

## Supporting Information

Additional supporting information can be found online in the Supporting Information section.

## Supporting information


**Supporting Information** Supporting Figure S1: Learning curve of the random forest model for predicting CVD visit volume. Supporting Figure S2: Predicted versus observed values of CVD visits based on random forest regression.

## Data Availability

Due to privacy and ethical restrictions, the individual‐level clinical records used in this study are not publicly available. However, the environmental and meteorological datasets used are publicly accessible through the links provided in the manuscript.
