# Supplementary material for: Short‐Term Interactive Effects of Temperature and Air Pollution on Cardiovascular Visits: A Machine‐Learning Study in Hohhot, China
Source: Cardiol Res Pract. 2026 Nov 10;2026:9938043. doi: 10.1155/crp/9938043 (PMC13752485; doi:10.1155/crp/9938043)
Supplement: Supplementary file 1 [file CRP-2026-9938043-s001.docx]

**Fig. S1** Learning curve of the random forest model for predicting CVD visits volume

The blue line represents the training set, and the red line represents the validation set.

**Fig. S2** Predicted versus Observed values of CVD visits based on Random Forest Regression
